# Multiple Myeloma and Vasculitic Neuropathy: An Unusual Presentation

**DOI:** 10.7759/cureus.13776

**Published:** 2021-03-09

**Authors:** Nabin Simkhada, Prakash Adhikari, Nisha Baral, Bishal Dhakal, Krish Mahat

**Affiliations:** 1 Internal Medicine, Nepalese Army Institute of Health Sciences, Kathmandu, NPL; 2 Internal Medicine, Piedmont Athens Regional Medical Center, Athens, USA; 3 Microbiology, Manipal College of Medical Sciences, Pokhara, NPL

**Keywords:** multiple myeloma, peripheral neuropathy, vasculitic neuropathy, neuropathy, monoclonal gammopathy

## Abstract

Multiple myeloma (MM) is a clonal proliferation of plasma cells in the bone marrow resulting in the production of paraproteins. It is more common in elderly adults and presents with nonspecific symptoms like bone pain, pathological fracture, fatigue, and signs of hypercalcemia. Peripheral neuropathy is an atypical presentation. We present a rare case of vasculitic neuropathy (VN) who was also diagnosed with MM at the same time. Nerve conduction study and biopsy showed findings suggestive of demyelinating VN. His serum protein electrophoresis and bone marrow aspirate were consistent with MM. The association between these two conditions remains understudied. So far there is no strong evidence suggesting an association between MM and VN. If VN was just coincidental or a presenting symptom of MM remains a question and warrants further studies.

## Introduction

Multiple myeloma (MM) is a monoclonal gammopathy characterized by the proliferation of clonal plasma cells within the bone marrow. The plasma cells exceed 10% of all bone marrow cells leading to an abnormal increase in clonal paraproteins which causes specific end-organ damage and acts as a disease marker [1]. It accounts for 1.8% of all new cancer cases and 2.1% of cancer deaths in the United States [2]. Clinical presentation is diverse; nonspecific symptoms like fatigue, loss of appetite, and weight loss being very common. Bone pain, pathological fracture, hypercalcemia leading to abdominal pain, thirst, excessive urine loss, and kidney failure are some typical presenting symptoms whereas, neuropathy is a very unusual presenting symptom [2].

In monoclonal gammopathy, the immunoglobulin type M (IgM) protein is commonly associated with peripheral neuropathy, but IgG and IgA neuropathies are rare [3]. The pathogenesis of neuropathy is thought to be a direct effect of M protein resulting in the demyelination process on the peripheral nerves [4]. But there is no strong evidence to support the causal relation between M protein and peripheral neuropathy. There is a need for a bigger study to better understand the association between MM and neuropathy. Lymphoproliferative disorders and myelodysplastic syndromes are common in patients with vasculitis [5,6]. Kapoulas et al. reported a case of MM presenting as a myeloperoxidase-antineutrophil cytoplasmic antibody (MPO-ANCA or p-ANCA) associated with microscopic polyangiitis [7]. Here we present a case of a patient who presented with symptoms of neuropathy and histopathology showed vasculitic neuropathy (VN), he was also diagnosed with MM at the same time.

## Case presentation

A 60-year-old Nepalese male with no known comorbidities presented to our ED with a complaint of weakness on bilateral upper and lower limbs. Weakness first started in the right upper limb, then on left upper limb, and then started on bilateral lower limbs and was associated with a tingling sensation. He reported intermittent fever and weight loss for two months. He denied a history of loss of consciousness, seizure, neck rigidity, joint stiffness, bowel, and bladder incontinence. He has no history of smoking tobacco and alcohol intake. His family history is unremarkable, and he has no known allergy to any medications.

His vital signs at the time of presentation were normal. General examination showed multiple crusted lesions over the lower back and bilaterally over the lower extremities. There was no sign of pallor, icterus, lymphadenopathy, clubbing, cyanosis, edema, and dehydration. Examination of abdomen, heart, lungs was unremarkable. Neurological examination revealed decreased muscle tone, absent deep tendon reflex, and power of 4/5 on bilateral upper and lower extremities. Wrist drop was present on the right upper limb. Sensory examination revealed distal paresthesia and the vibratory sensation was decreased in bilateral lower limbs.

Complete blood count showed leukocytosis with 56% eosinophils and peripheral blood smear showed peripheral eosinophilia. The rest of the complete blood count and comprehensive metabolic panel are shown in Table 1.

**Table 1:** Complete blood count and comprehensive metabolic panel ALT- Alanine transaminase; AST- Aspartate transaminase; BUN: Blood urea nitrogen; D-bilirubin: Direct bilirubin; T-bilirubin: Total bilirubin.

Complete Blood Count	Comprehensive Metabolic Panel
White blood cell: 16,000/µL	Sodium: 136 mmol/L
Hemoglobin: 14.1 g/dl	Potassium: 3.8 mmol/L
Platelets: 290,000/µL	BUN: 74 mg/dl
Neutrophil: 30%	Creatinine: 7.7 mg/dl
Lymphocytes: 10%	Blood sugar: 135mg/dl
Monocytes: 4%	Alkaline phosphatase: 59 U/L
Eosinophils: 56%	AST: 35 U/L
	ALT: 30 U/L
	T-bilirubin: 0.8 mg/dl
	D-bilirubin: 0.2 mg/dl

**Figure 1:**
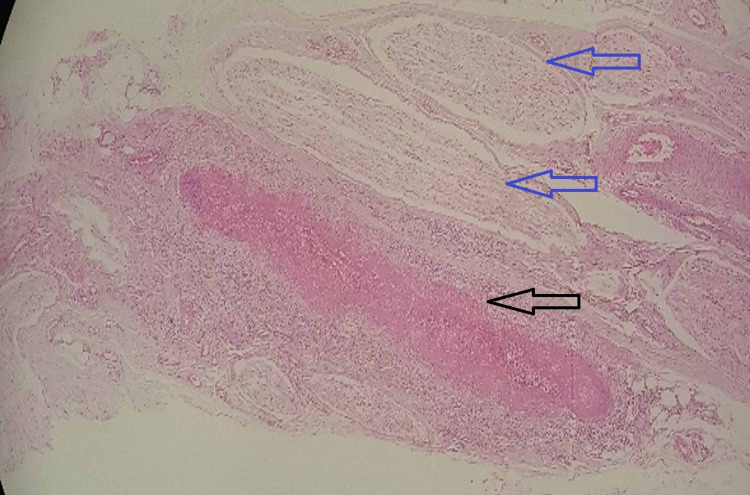
Sural nerve biopsy. Blue arrows showing nerve bundle; black arrow showing blood vessel with fibrinoid necrosis and inflammatory infiltrate within the vessel wall

The prothrombin time and international normalized ratio were normal. The serological tests for HIV, Hepatitis B, and C were nonreactive. The cerebrospinal fluid (CSF) analysis revealed glucose of 64 mg/dl, protein of 36.7 mg/dl, and total leucocyte count of 25 cells/cu mm. The proteinase-3 antineutrophil cytoplasmic antibody (PR3-ANCA or c-ANCA) and p-ANCA were positive. Nerve conduction study showed demyelinating polyneuropathy of bilateral median and ulnar nerves on upper extremities and bilateral common peroneal and posterior tibial nerves on lower extremities. A sural nerve biopsy was performed and findings were suggestive of VN (Figure 1).

The bone marrow aspirate showed normocellular bone marrow with 12% of plasma cells. Serum protein electrophoresis (SPEP) showed hypergammaglobulinemia, findings consistent with MM. The result of SPEP is shown in Table 2.

**Table 2:** Table showing result of serum protein electrophoresis (SPEP)

Test Details	Results	Reference Range
Total Protein	7.1 g/dL	6.4-8.3
Serum Albumin	2.27 g/dL	3.57-5.42
Alpha 1 Globulin	0.43 g/dL	0.19-0.40
Alpha 2 Globulin	1.09 g/dL	0.45-0.96
Beta Globulin	0.66 g/dL	0.30-0.59
Gamma Globulin	2.64 g/dL	0.71-1.54
Albumin/Globulin Ratio	0.47	1.0-2.2
Free Kappa Light Chain	331.22 mg/L	3.3-19.4
Free Lambda Light Chain	179.7 mg/L	5.7-26.3
Free Kappa/Lambda Ratio	1.84	0.26-1.65

Our patient was diagnosed as MM with VN. He was treated with intravenous methylprednisolone one gm for five days. Then he was started on oral prednisolone of 60 mg once daily to be tapered gradually over 18 months. The definitive chemotherapy with bortezomib, dexamethasone, and thalidomide regimen was planned, and the induction phase was initiated.

## Discussion

MM is a neoplasm of plasma cells that is characterized by clonal plasma cell proliferation and the formation of monoclonal paraprotein which causes specific end-organ damage. It is a part of a spectrum of monoclonal gammopathy. It occurs predominantly in a population above 70 years. The etiology is not clear, but it is thought to be related to genetic abnormalities in oncogenes such as c-Myc, NRAS and KRAS. There is evidence that suggests other factors like drinking alcohol, obesity, environmental factors, and radiation exposure associated with the development of MM. [8]. The diagnosis is based on National Comprehensive Cancer Network (NCCN) guidelines diagnostic criteria which include [9]:

Bone marrow clonal plasma cells ≥ 10% and one or more myeloma defining events including:

- Serum calcium ≥1 mg/dL higher than the upper limit of the normal range or >11 mg/dL

- Renal insufficiency (creatinine >2 mg/dL)

- Anemia (hemoglobin <10 g/dL or hemoglobin <2 g/dL below the lower limit of normal)

- One or more osteolytic bone lesions on skeletal imaging

The clinical signs and symptoms of the disease are very diverse, patient may present without symptoms or with severe illness. Common symptoms and signs include anemia, bone pain, derangement in kidney function, generalized weakness, hypercalcemia, and weight loss [10]. Neurological manifestation is not uncommon and is usually present with symptoms of spinal cord compression either from bone fragment due to fracture of the vertebral body or from extramedullary plasmacytoma. Hyper viscosity due to IgM can also present as confusion and other neurological signs [11]. Peripheral neuropathy is rare at the time of initial diagnosis though it is common with other plasma cell abnormalities. Light chain amyloidosis (AL) and osteosclerotic myeloma (also known as POEMS syndrome- polyneuropathy, organomegaly, endocrinopathy, monoclonal protein and skin changes) are associated with progressive chronic demyelinating sensorimotor polyneuropathy [4].

The presence of neuropathy in MM is poorly understood and it is not clear if there is any association or if it is coincidental. Some studies showed a presence of neuropathy in patients with monoclonal gammopathy and vice versa. One of the studies showed 3% of patients with monoclonal gammopathy had peripheral neuropathy [12]. Another study on patients with peripheral neuropathy detected 5% of patients with plasma cell dyscrasia [13]. Neuropathies associated with monoclonal gammopathy have been shown to be more common with IgM compared to IgG and IgA [14]. It has been thought that M proteins can directly affect peripheral nerves and can result in the demyelination process [4]. But there is a lack of sufficient evidence to support this and many researchers think the presence of peripheral neuropathy may be coincidental.

In addition to MM, our patient had peripheral neuropathy with evidence of ANCA (both p-ANCA and c-ANCA) associated vasculitis hence he was diagnosed with VN. Our patient had peripheral eosinophilia which made us think of eosinophilic granulomatosis with polyangiitis in the beginning, but he did not meet the criteria as there was no systemic involvement (no history of asthma, pulmonary infiltrate, or sinus disease). The renal impairment in our patient could be itself from MM-associated immune deposit and cast nephropathy. But it could also be from ANCA-associated vasculitis due to mononuclear cell infiltration and destruction of the vessel wall. Due to a lack of expertise, we could not perform a renal biopsy to confirm the exact etiology of renal insufficiency.

## Conclusions

MM is a common condition in the elderly population with very diverse presenting symptoms. In this case, the patient presented with peripheral VN and he was also diagnosed with MM. Whether peripheral VN is associated with MM or if it is coincidental is not clear. Further studies are needed to show their association. This case report highlights the fact that nonspecific clinical presentation should not be overlooked in an elderly population, the presentation could be a clue to some underlying neoplasm.
